# Digital image processing: A new tool for morphological measurements of freshwater turtles under rehabilitation

**DOI:** 10.1371/journal.pone.0300253

**Published:** 2024-03-14

**Authors:** Ashish Kumar Panda, Vikas Verma, Anupam Srivastav, Ruchi Badola, Syed Ainul Hussain

**Affiliations:** 1 Ganga Aqualife Conservation and Monitoring Centre, Wildlife Institute of India, Chandrabani, Dehra Dun, Uttarakhand, India; 2 Academy of Scientific and Innovative Research (AcSIR), Ghaziabad, Uttar Pradesh, India; University of Basrah, IRAQ

## Abstract

Freshwater fauna is facing an uphill task for survival in the Ganga Basin, India, due to a range of factors causing habitat degradation and fragmentation, necessitating conservation interventions. As part of the ongoing efforts to conserve the freshwater fauna of the Basin, we are working on rehabilitating rescued freshwater chelonians. We carry out various interventions to restore rescued individuals to an apparent state of fitness for their release in suitable natural habitats. Morphometric measurements are crucial to managing captive wild animals for assessing their growth and well-being. Measurements are made using manual methods like vernier caliper that are prone to observer error experience and require handling the specimens for extended periods. Digital imaging technology is rapidly progressing at a fast pace and with the advancement of technology. We acquired images of turtles using smartphones along with manual morphometric measurements using vernier calipers of the straight carapace length and straight carapace width. The images were subsequently processed using ImageJ, a freeware and compared with manual morphometric measurements. A significant decrease in the time spent in carrying out morphometric measurements was observed in our study. The difference in error in measurements was, however, not significant. A probable cause for this may have been the extensive experience of the personnel carrying out the measurements using vernier caliper. Digital image processing technology can cause a significant reduction in the stress of the animals exposed to handling during measurements, thereby improving their welfare. Additionally, this can be used in the field to carry out morphometric measurements of free-ranging individuals, where it is often difficult to capture individuals, and challenges are faced in obtaining permission to capture specimens.

## Introduction

Freshwater bodies are the lifeline of human civilizations [1]. However, usable freshwater found in lakes, ponds, rivers, etc., is less than 1% of the total water volume on our planet [2]. These habitats support a rich biodiversity, essential for maintaining ecosystem functioning and services [3]. This diversity is increasingly threatened by the extraction of water for various uses, the development of linear infrastructure and unsustainable resource extraction practices [3, 4]. Rivers and other freshwater linear features are frequently used as boundaries for delineating Protected Areas [5]; however, they themselves are rarely identified as conservation targets in their own right [1], leading to this rapid loss of biodiversity.

A total of 436 chelonian species have been recorded worldwide, and this includes 336 modern species and 100 extinct Pleistocene and Holocene taxa, with 69 (57.0%) of the modern species having become extinct since the start of the Pleistocene [6]. They are keystone species of their habitats, benefitting other animals and plants through a complex web of interactions for the healthy functioning of ecosystems [7]. Yet they are also one of the most threatened groups of animals [3] and are facing an extensive illegal trade [8] that further undermines their survival. The IUCN Red-list of Threatened Species places 63% of chelonians under various threat categories and 10% as critically endangered.

The Indian subcontinent, with its rich aquatic biodiversity that is supported in both inland and marine areas due to its unique biogeography along with varied topography and climate [9], is one of the 17 mega-diverse countries considering turtle richness [10, 11]. The diversity of its chelonian fauna is reflected both in the freshwater and marine aquatic ecosystems. The stressors stated earlier directly and indirectly impact freshwater organisms, including chelonians, at various levels by altering their habitat and community structure [12], and the judicious rehabilitation of rescued individuals can help in achieving conservation goals.

Strategies used include the relocation of nests vulnerable to predation and/or inundation to safer areas *in-situ* and captive rearing facilities for head-starting programs. Followed by the release of individuals reaching a size that can ensure their survival into suitable habitats [13] and the use of rescued animals after restoration to fitness in captive facilities for relocation/ repatriation to suitable habitats [14].

The likelihood of rescued animals contributing to conservation goals is dependent on the condition of the animals, release site characteristics and protocols adopted for rehabilitation [15]. While a body of information exists on the best practices to be adopted for the management of rehabilitation of wild terrestrial mammals in India, limited information exists on the management of rescued aquatic fauna [16]. The condition of rescued animals prior to and during rehabilitation is perhaps one of the best indicators of the likelihood of success of release efforts. It can be assessed by a comparison of body mass with size (usually linear measurement) [15].

Morphometric studies on turtles have primarily relied on measurements using vernier calipers and flexible tapes [17–19]. These measurements are, however, prone to observer and equipment errors. The observer requires longer times for processing and may cause stress to the animals due to handling [20–23]. The advent of digital imaging technology has created an opportunity for carrying out morphometric measurements using digital tools. The evolution of digital imaging technology and collaborative studies between digital imaging experts and life scientists has paved the way for this use [24]. An extensive body of literature exists on biomedical image processing using digital tools [25–27]. One of these, the ImageJ Project [28] of the National Institute of Health, USA, is an open-source software of relevance to life scientists for carrying out morphometric measurements of whole organisms.

Morphometric analysis serves as a crucial tool because it allows for precise measurements due to the unique body structures. Such data on morphometric variation have been recorded in select Chelonian studies [29]. Morphometric measurements play an essential role in the assessment of growth patterns and the physiological state of individuals [16, 30] and form the basis for evaluating the fitness status of individuals in rescue situations where other diagnostic tools may not be available [31]. Digital image processing is of particular relevance in the management of rescued chelonians as the technique is non-invasive and enables the rapid processing of a large number of animals that are often received in rescue [31]. Accordingly, we undertook to assess the efficiency of morphological measurement using vernier calipers and digital measurements.

## Materials and methods

### Study area

**Fig 1:**
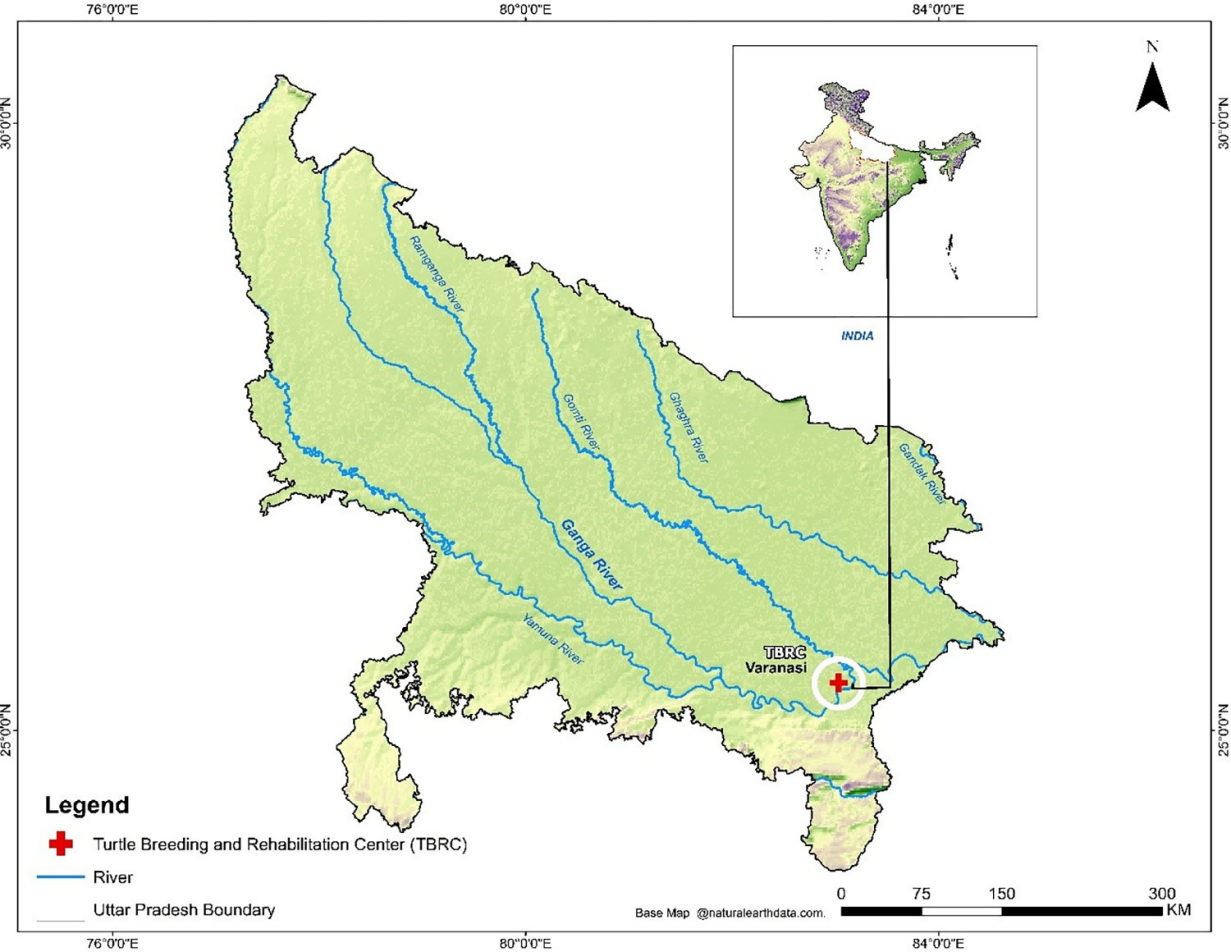
The turtle breeding and rehabilitation center (TBRC) Sarnath, Varanasi, India. Base map source- Naturalearth.

The present study has been conducted in the Turtle Breeding and Rehabilitation Center (TBRC) at Sarnath, Varanasi, India (Fig 1). TBRC was established in 1989 after the declaration of Turtle Sanctuary at Varanasi as part of the Ganga Action Plan. TBRC was an *ex-situ* adjunct to *in-situ* conservation efforts as part of the Ganga Action Plan. It was created to reestablish the population of the Indian Soft-shell Turtle (*Nilssonia gangetica*), a species that has faced sharp declines. Since its inception in 1989 as a rearing and release centre for the Ganges Softshell Turtle, the centre has diversified into a rehabilitation facility for rescued freshwater turtles and rearing of hatchlings of rescued eggs for multiple species of freshwater turtles [31].

### Materials

**Fig 2:**
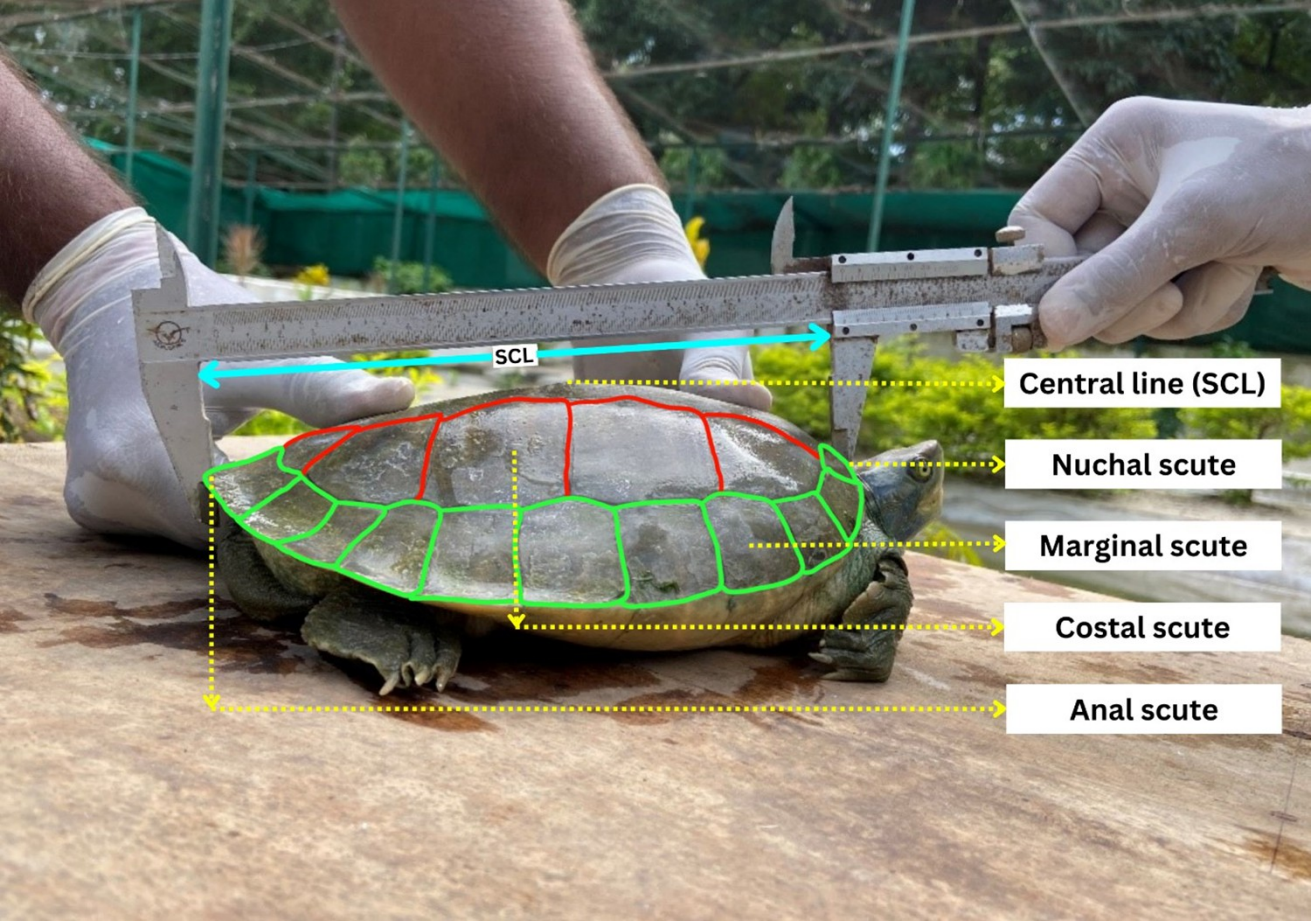
Morphometric measurements (e.g. SCL) were obtained using the vernier caliper of *Batagur kachuga*.

The present study was carried out during 2021 (April) monthly morphometry process to compare the two morphometry techniques. The turtles housed at TBRC, Varanasi, were used to perform the test (*Batagur dhongoka*, *Batagur kachuga*, *Lissemys punctata*, *Hardella thurjii*, *Melanochelys tricarinata*, *Pangshura smithii* and *Pangshura tecta)*. Standardized protocols for recording morphological measurements were followed based on existing guidelines [17–19]. Measurements were taken using vernier calipers: a straight line of straight carapace length (SCL) and straight carapace width (SCW) (Fig 2**).** Sex data was not collected as-mixed age class, and species individuals were sampled, and sexual characters were poorly manifested in the juvenile and subadult stages, making identification of sex challenging in these individuals.

#### Vernier caliper

**Fig 3:**
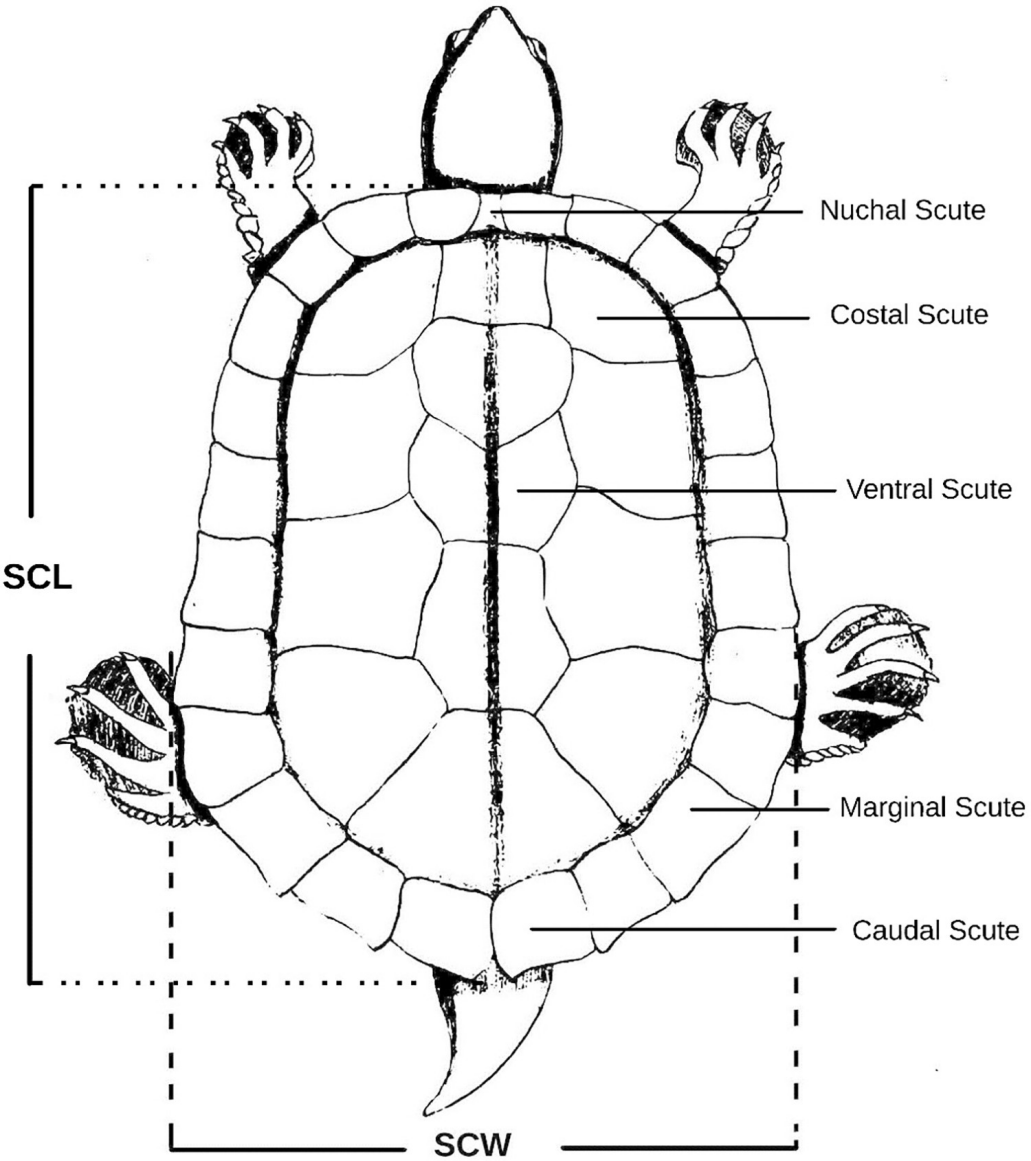
Morphometric measurements of the carapace of *Batagur dhongoka* (Illustration) showing the straight carapace length (SCL) and Straight carapace width (SCW).

*Carapace length*. The measurement of straight carapace length (SCL) involved determining the distance from the front edge of the carapace (Nuchal scute) to the rear tip of the carapace (supra-caudal scute), as illustrated in Fig 3. Both the anterior and posterior measurement points were situated on the same side of the carapace (along the median ridge). To maintain consistency, the side yielding the greater SCL measurement was selected [32, 33], as depicted in Fig 3.

*Carapace width*. Straight carapace width (SCW) was determined at its widest point using vernier caliper, as depicted in Fig 3, due to the unavailability of specific anatomical reference points. Special attention was given to maintaining a consistent orientation of the turtle during SCW measurements, particularly with juvenile turtles, to avoid introducing additional variability. It’s worth noting that when a turtle lies on its carapace (plastron up), the mass of the turtle can exert pressure and widen the carapace, impacting the width measurement. Additionally, carapace width may vary as the turtle breathes in and out. To ensure uniformity, SCW measurements were exclusively taken with the turtle in a lateral recumbent position, as illustrated in Fig 3.

#### ImageJ Software and data processing

**Fig 4:**
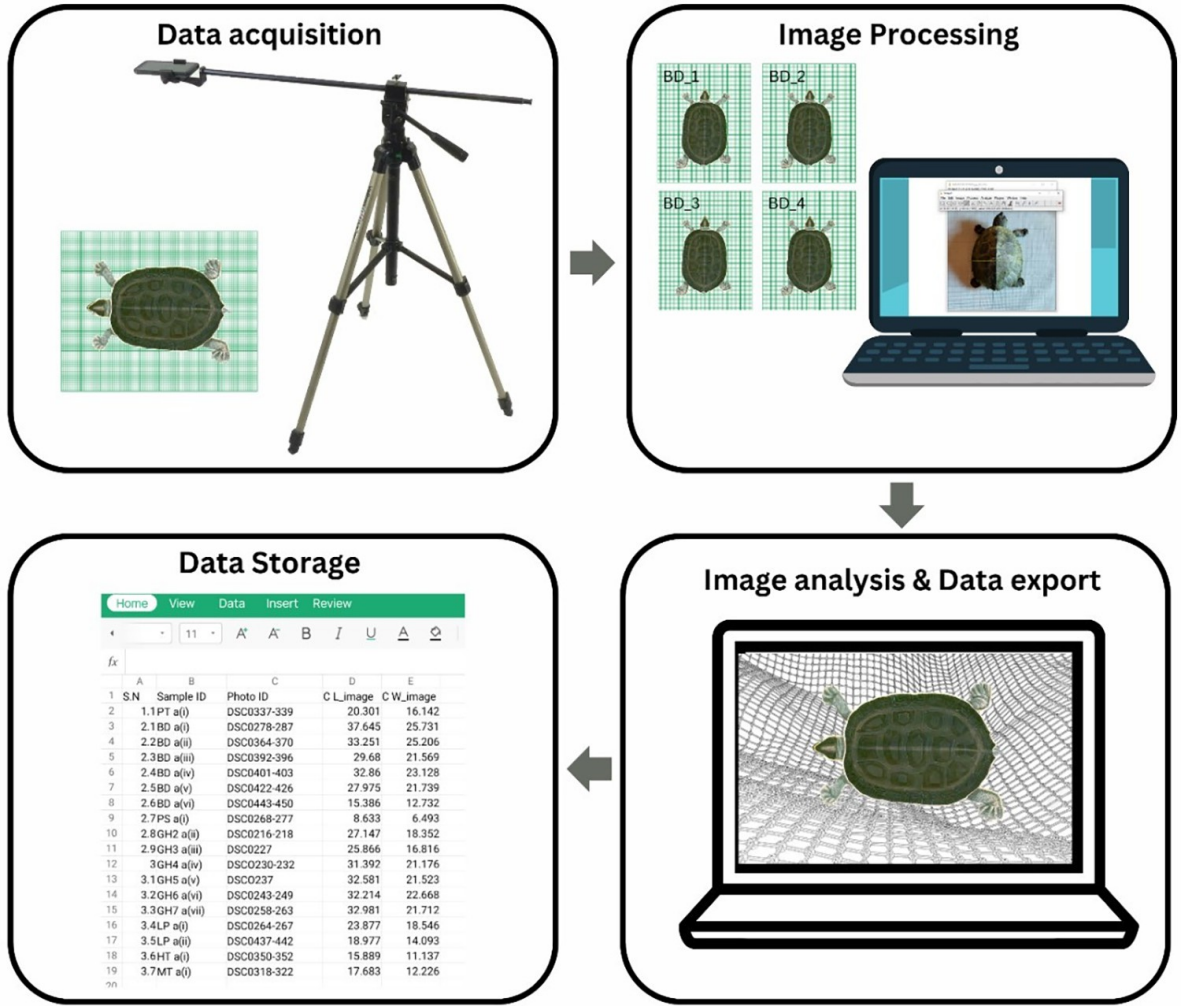
Morphometry data acquisition, analysis and storage using ImageJ.

**Fig 5:**
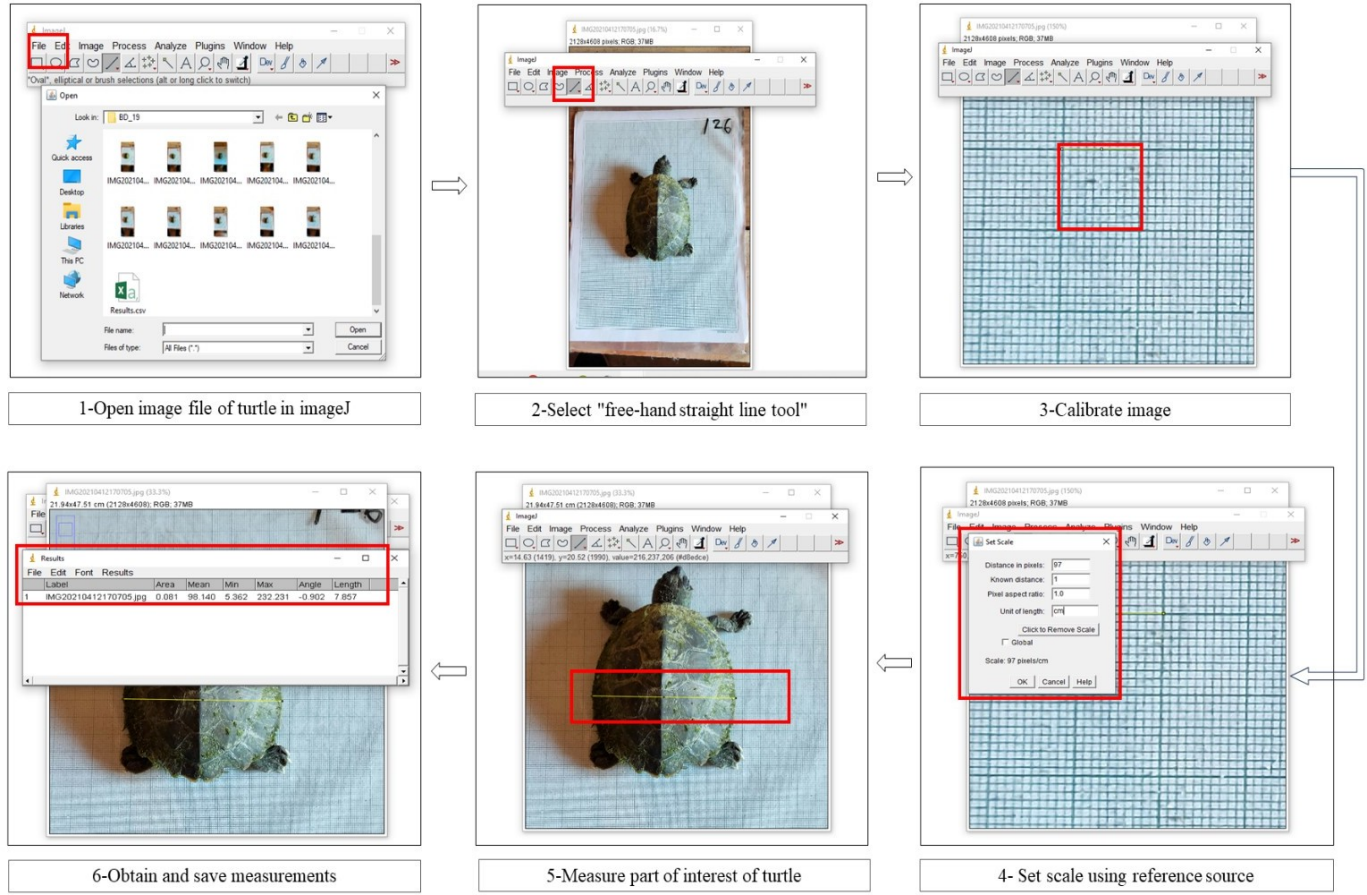
ImageJ step-by-step method for the digital morphometry process.

**Fig 6:**
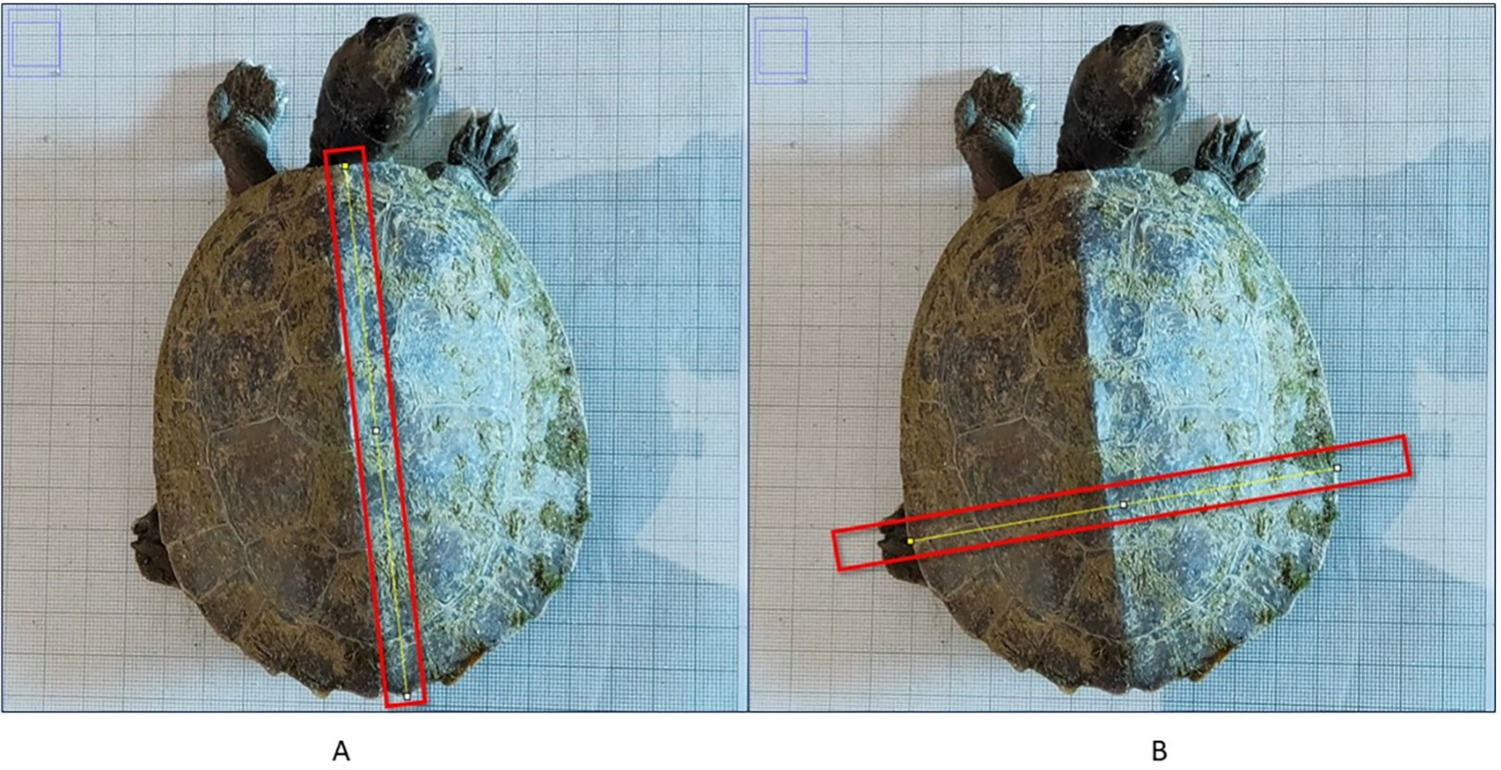
Morphometry of Carapace of *Batagur dhongoka* using ImageJ (A) SCL-straight carapace length and (B) SCW-straight carapace width.

In the present study, we used ImageJ software [34] for digital measurements. Turtles were placed on the table on water-resistant graph paper as a background to capture their images. We identified the individuals by using the notch-cutting technique described by Cagle [35]. Additionally, we marked the graph papers where the photos were taken with unique numbers using a temporary marker pen. We also assigned photo IDs for reference to cross-validate the Photo ID, Graphsheet ID, and Turtle ID. These images on the graph paper are used to calibrate the reference scale in ImageJ software [34] (Figs 4 and 5**)**. All photographs were captured on a smartphone, which was placed on a tripod. In our initial study, we tested various DSLR and point-and-shoot (fixed lenses) cameras and found minimal differences in accuracy (±0.10cm). Smartphones stood out as the preferred option due to their widespread availability and precision. After the image folder is selected, each image is scaled, and their carapace length and width are measured in ImageJ (Fig 6**).**

ImageJ is a free Java-based image processing and analysis programme. It is available as an applet online or as a download for Windows, Mac OS X, and Linux. It supports image stacks and various image types (8-bit, 16-bit, and 32-bit) and formats (e.g., TIFF, GIF, JPEG). ImageJ is multithreaded, which allows for concurrent operations such as image file reading. It is, in essence, a versatile tool for viewing, editing, analysing, and processing images that was inspired by NIH Image for Macintosh [34].

#### Ethics statement

All procedures conducted in this study were carried out during the routine monthly health check-ups and standard husbandry practices under the supervision of the veterinary officer. The protocols adopted were designed to ensure the well-being of the freshwater turtles and did not involve any pain, suffering, distress, or lasting harm. We received the necessary permission and ethical clearance from the Uttar Pradesh Forest Department (UPFD) for the management of animals at the Turtle Breeding and Rehabilitation Center in Sarnath [Permit no. 1101/23/02/2012(G) dated 27/10/2016], with an extension no. [1854/23/02/2012(G) dated 02/12/2022]. The project team provides technical support in the UPFD rehabilitation efforts. The work presented here is a part of this endeavour. This study was approved by the Ministry of Jal Shakti, Government of India, vide letter (No. B-02/2015-16/1259/NMCG-WIIPROPOSAL) with an extension (No. B-03/2015-16/1077/NMCG).

#### Statistical analysis

Statistical evaluation included the assessment of errors and correlations between measurements obtained from both methods. The error was quantified by calculating the absolute difference between the digital measurements and the corresponding physical measurements obtained using vernier calipers. The correlation between the vernier calliper and ImageJ digital measurement method was tested by running a Spearman rank correlation test in Rstudio. The precision of both methods was tested using the paired sample *t*-test for means, which assumes that the true mean difference between the paired samples was zero [36], with the test run in Rstudio. To evaluate the accuracy of the model, the root mean squared error (RMSE) and mean absolute error (MAE) [37] were calculated using the “MLmetrics” package in Rstudio. Before running the test, each value obtained from both methods was formatted according to the test. The test was run by comparing the distance value obtained from the vernier caliper and ImageJ digital measurement.


$$RMSE=\sqrt{\bar{{\left(f-0\right)}^{2}}}MAE=\frac{1}{n}\sum_{i=1}^{n}\left|x_{i}-x\right|$$

**Where**:

- f = forecasts (expected values or unknown results),
- o = observed values (known results).
- n = the number of errors,
- Σ = summation symbol (which means “add them all up”),
- |x_i_ –x| = the absolute errors.

## Results

**Fig 7:**
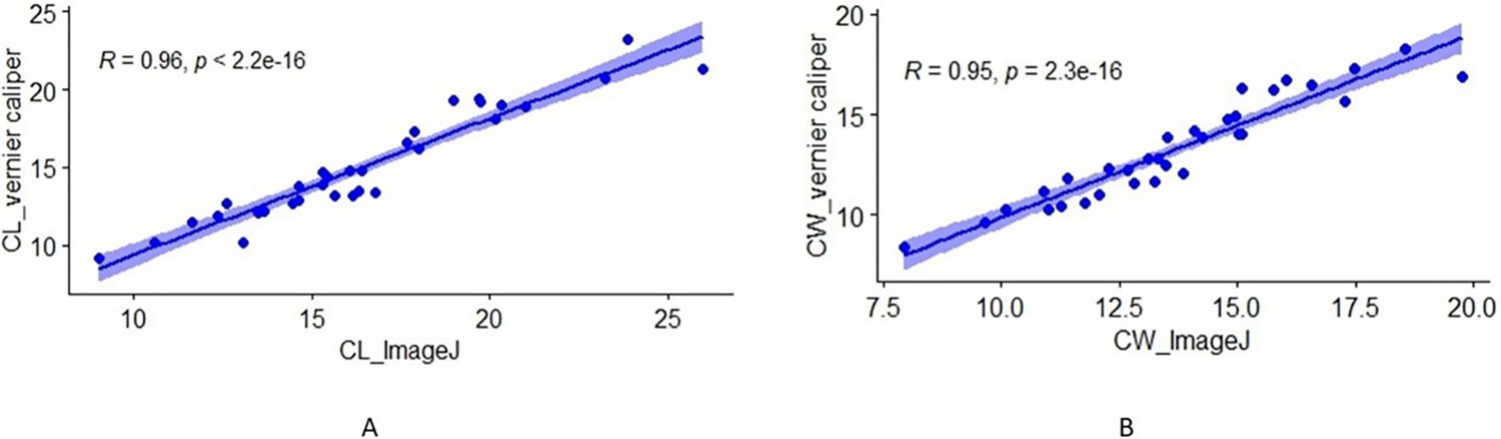
The correlation coefficient between the vernier caliper and the ImageJ for both the measurement of (A) carapace length and (B) carapace width.

A total of 172 individuals, including 42 specimens of *Batgur kachuga* (adult), 82 of *Batagur dhongaka* (Juvenile), 32 of *Lissemys punctata* (adult*)*, 6 of *Batagur dhongoka* (Adult), 6 of *Geoclemys hamiltonii* (Adult) and 4 of mixed specimens (*Pangshura tecta*, *Pangshura smithii*, *Hardella thurjii*, *Melanochelys tricarinata*) from the Turtle Breeding and Rehabilitation Center (TBRC) at Sarnath were analyzed to compare the variance between the two techniques. Morphometric measurements (SCL, SCW and PL) were carried out using both the ImageJ and the vernier caliper. Table 1 presents the t-test outcomes used for evaluating the precision of each respective method. Based on P-values, all the turtles show high precision in both the vernier caliper and ImageJ methods. Table 2 shows values obtained using the t-test paired two samples for means to evaluate the measurement obtained with the vernier caliper and the ones obtained from the ImageJ. The repeatability test yielded a correlation coefficient indicating high repeatability for both methods (Fig 7). Furthermore, there is no apparent indication of any association between the measured length and the accompanying measurement error (Fig 7). Fig 7 indicates a strong correlation between measurements taken with vernier calipers and those obtained through ImageJ software, R^2^ = 1.

**Table 1:** Carapace dimensions and errors with the vernier caliper and the ImageJ method.

Error in Measurements (cm)		Species								
		*Batagur dhongoka* (Adult)	*Batagur dhongoka* (Juvenile)	*Batagur kachuga* (Adult)	*Lissemys punctata* (Adult)	*Geoclemys hamiltonii* (Adult)	*Pangshura tecta*	*Pangshura smithii*	*Hardella thurjii*	*Melanochelys tricarinata*
Carapace Width	Average ± SD	0.41 ± 0.29	0.27 ± 0.21	0.45 ± 0.3	0.7 ± 0.64	0.52 ± 0.29	0.16	0.01	0.16	0.77
	Minimum	0.04	0.01	0.02	0.01	0.18	-	-	-	-
	Maximum	0.77	0.8	1.05	2.86	0.91	-	-	-	-
Carapace Length	Average ± SD	0.69 ± 0.41	0.48 ± 0.29	0.84 ± 0.42	1.46 ± 1.09	0.48 ± 0.44	1.2	0.33	0.31	0.38
	Minimum	0.12	0.01	0.16	0.1	0.03	-	-	-	-
	Maximum	1.25	1.72	2.1	4.69	0.98	-	-	-	-

**Table 2:** P-values obtained using the t-test paired two samples for means using vernier calipers and ImageJ measurements.

Species	SCL			SCW		
	P	95% confidence interval		P	95% confidence interval	
*Batagur dhongoka* Juvenile	2.20E-16	0.377918	0.5025694	2.02E-14	0.191863	0.2962837
*Batagur kachuga* adult	4.33E-16	0.710431	0.9726644	1.72E-10	0.327088	0.5331976
*Lissemys punctata* adult	6.73E-08	1.085887	1.010752	0.006675	0.129505	0.7380579
*Batagur dhongoka* adult	-.9695567	0.8352234	0.5971344	0.8821	8.56E-01	-0.5288011
*Geoclemys hamiltonii* adult	0.08782	-0.09213	0.9524624	0.006592	0.222732	0.8262676

In our study, we evaluated the efficiency of two methods for measuring morphometric traits in turtles: vernier caliper and ImageJ. In addition to measuring the accuracy of the two methods, we also calculated the time and animal handling duration required to complete both processes. Our results showed that the vernier caliper approach took an average of 3.23 minutes (203 s) to complete, whereas the ImageJ method took only 48 seconds to measure all of the morphometric traits. This indicates that the handling time in digital morphometry was reduced by 96.23%, making it a more efficient and time-saving method compared to the traditional vernier caliper approach. It’s important to note that the handling duration in softshell turtles and adult turtles was slightly longer, accounting for about 18% of the total time required for the morphometry procedure. In contrast, hardshell turtles took significantly less time to complete both procedures (Table 3).

**Table 3:** Time is taken for measurements using the vernier caliper and the ImageJ methods.

Duration of the measurement (Seconds)		Species								
		*Batagur dhongoka* (Adult)	*Batagur dhongoka* (Juvenile)	*Batagur kachuga* (Adult)	*Lissemys punctata* (Adult)	*Geoclemys hamiltonii* (Adult)	*Pangshura tecta*	*Pangshura smithii*	*Hardella thurjii*	*Melanochelys tricarinata*
Vernier Caliper	Average ± SD	154± 7.07	153 ± 1.41	206.5± 96.87	177.5 ± 45.96	201.5 ± 108.18	-	-	-	-
ImageJ	Average ± SD	52± 1.41	50.5 ± 4.94	43.5 ± 17.67	46 ± 2.82	47.5 ± 2.12	-	-	-	-

**Fig 8:**
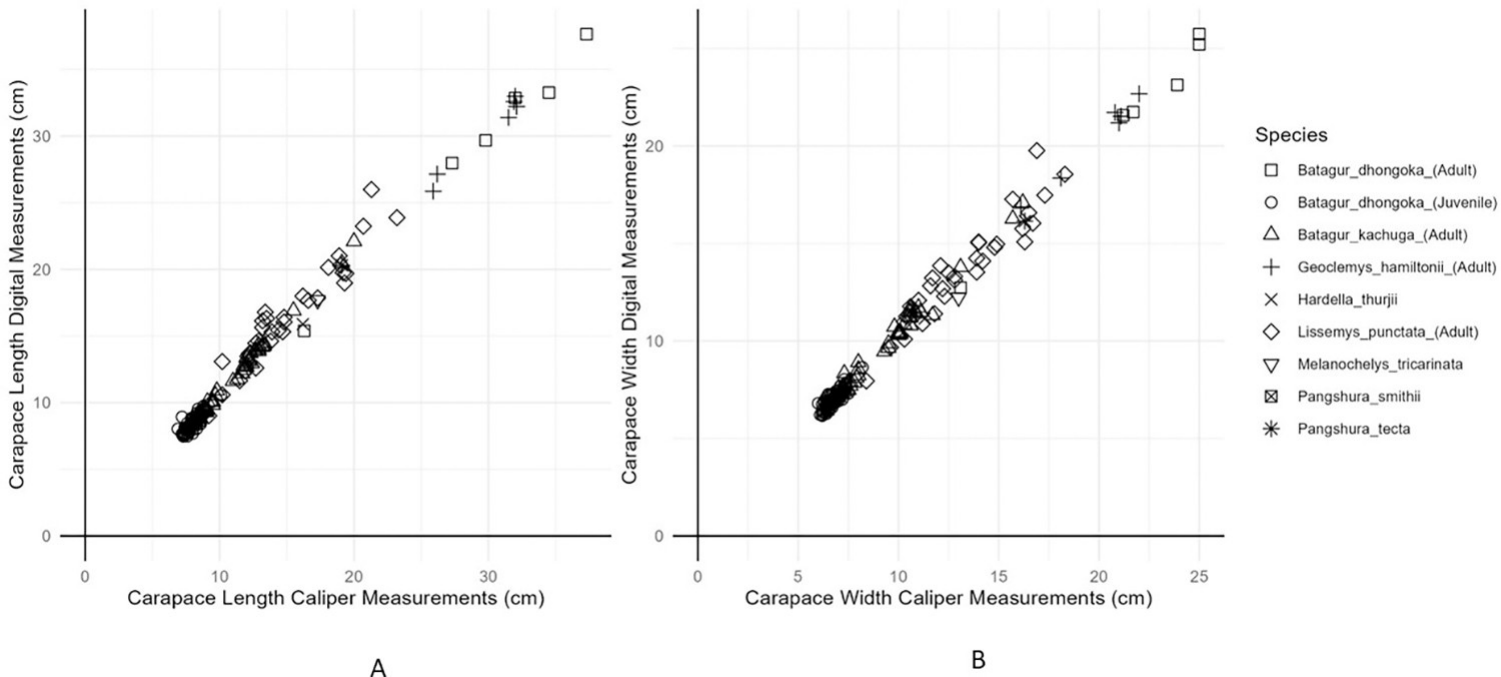
Relationship between measurements from the digital image processing (ImageJ) and those obtained with the vernier caliper (A) straight carapace length (B) straight carapace width. (Triangles, squares, circles, and other symbols indicate different turtle species).

**Fig 9:**
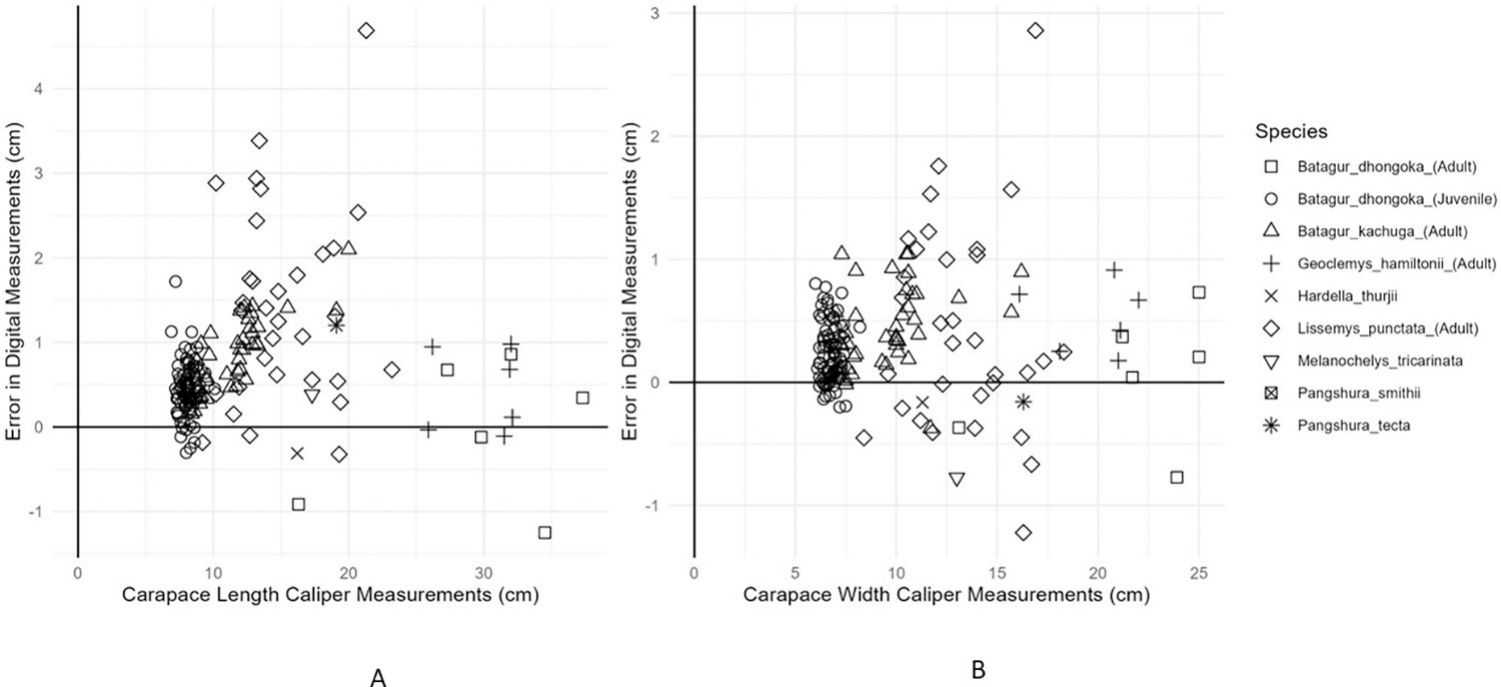
Relationship between the statistical error due to each method and the length and width measurement (A) Straight Carapace length (B) Straight Carapace Width. (Each turtle species is represented by a unique shape).

The photographic measurements for the estimation of carapace length and width were calculated with an approximate root mean square error of 0.71 and 0.32 and mean absolute error of 0.76 and 0.41, respectively. As shown in (Fig 8), a graphical representation of the measurements made by both methods and the error in estimating the carapace length and width (Fig 9).

## Discussion

The present study is aimed at developing a rapid method for morphometric measurements of turtles in our care to facilitate in decision support for planning and undertaking interventions during rehabilitation. For this we compared the precision and time taken in the two methods, ImageJ and vernier caliper, for measuring the carapace length and width of animals. Our results indicated that both methods had almost similar precision for measuring the carapace length and width of the animals, with no significant difference between the measurements obtained from the two methods (Figs 8 and 9 and Table 2). The minor differences in precision were attributed to less precise camera calibration. The repeatability of both methods was high, indicating that both methods are reliable (Fig 7 and Table 1).

Carrying out morphometric measurements using ImageJ involved the use of existing smartphones present with the researchers and the outcomes of the measurements made were unaffected by the different instruments used. The digital method, ImageJ, has the advantage of being minimally invasive, avoiding additional stress on the animals, and automatically calculating corresponding points everywhere on the carapace. This feature makes it especially suitable for use in field conditions where it may not always be possible to fix the observer and device positions.

An additional advantage of digital image processing using ImageJ is the reduction in handling time, which results in reducing the stress the animals are exposed to while carrying out morphometric measurements (Table 3 and Fig 6). The feature is of particular importance in the rehabilitation of conservation-dependent species where the survival of every individual and its contribution to the growth of the population are of great importance for the sustained survival of the species [16]. In addition to its non-invasive nature, the ImageJ method also offers the advantage of automatic detection of corresponding landmark points on a turtle’s carapace that are then used for measurements [38]. This is accomplished using a process known as landmark analysis, which involves the identification of specific points on an animal’s body and the measurement of the distance between those points [39]. By using this method, researchers can quickly and accurately analyze morphological features of interest [23, 40–42].

Moreover, ImageJ is capable of generating a variety of statistical analyses, including t-tests, ANOVA, and linear regression [43, 44]. These statistical analyses can be used to compare different groups of animals, test for differences in size, and investigate the relationship between morphological traits and other variables such as age or sex [45–47]. These analyses can be particularly useful in studies that aim to identify morphological differences between different populations of animals or to assess the effects of environmental factors on animal morphology [43, 44, 48, 49].

Various morphometrics software options, such as MorphoJ, GMTP, and EFA, were also available for free, but all work on either outline-based or landmark-based methodologies [50–52]. These alternatives required approximately 10 minutes to analyze a single specimen. In contrast, ImageJ, renowned for its efficiency, completes analysis in an average of just 30 seconds [53–55]. Additionally, the other software options necessitated a proper photographic setup and high-quality camera equipment for image acquisition, potentially inducing stress in the animals and raising the cost of the measurements. Prioritizing the well-being of the animals in the rehabilitation center, given their large numbers, led to the decision to use ImageJ due to its time efficiency, ease of measurement and less invasive nature.

The utility of ImageJ in analyzing animal morphology has been well-demonstrated in prior research. Maulida et al. [56] conducted a study that utilized ImageJ for the analysis of Hawksbill Turtle carapaces. Their findings highlighted the efficiency and accuracy of ImageJ in assessing carapace curvature and various other morphological attributes, including size and shape. They concluded that ImageJ serves as a valuable resource for examining turtle morphology, particularly in fieldwork scenarios where studying animals in their natural habitats is essential.

In a similar vein, Zhang et al. [57] conducted a study that further underscores the effectiveness of ImageJ for analyzing animal morphology. Their study employed ImageJ to evaluate the morphological characteristics of crab carapaces. The results demonstrated that ImageJ offers a robust tool for investigating carapace curvature and shape. These studies reinforce the reliability and versatility of ImageJ in analyzing animal morphology, which is highly relevant to our work and aligns with our findings regarding the benefits of using ImageJ for turtle morphometric measurements.

Our findings agree with the studies discussed above that have examined the comparison between digital and traditional methods for assessing animal morphology. For instance, Mojekwu and Anumudu [58] conducted a study where they compared digital photogrammetry to traditional measurements for evaluating fish morphological traits. Their results demonstrated that digital photogrammetry was more accurate and less invasive than traditional methods. Likewise, Samara et al. [59] found that ImageJ-based modelling served as an effective and cost-efficient tool for studying human tissue.

Graph paper use in our study may have contributed to the consistent performance of ImageJ, as the grid provided a reference point that benefitted both observers and the software. Nevertheless, it’s crucial to acknowledge that further research is warranted to explore how these methods perform when applied to different animal species, various morphological traits, and under diverse measurement conditions. This insight further underscores the relevance of our study, as it corroborates the advantages of using digital methods like ImageJ in animal morphology research and emphasizes the potential for widespread applicability across various research contexts.

The research conducted by Stayton [60] focused on measuring the carapace shape and hydrodynamic analyses of turtles carcasses using the ImageJ. They concluded that ImageJ was more accurate and reliable than traditional measurement methods and capable of extracting a more extensive range of information from carapace shape data. This alignment underscores the consistent advantages of using ImageJ in studying animal morphology and reinforces the credibility of our results.

Similarly, the research by Schmidt and Schaefer [61], which compared the accuracy of ImageJ and traditional measurement methods for studying the body size of fish, has significant relevance to our study. Their results corroborate our findings, showing that ImageJ provided accurate and less error-prone measurements while enabling the extraction of additional information. This parallel strengthens the argument for the applicability and benefits of ImageJ in animal morphology research, further emphasizing the significance of our work in this context.

The study conducted by Llewelyn et al. [62], which employed the ImageJ method to investigate the morphology of lizard skulls, holds considerable relevance to our study. Their findings, indicating that the ImageJ method provided accurate results and was more time-efficient than traditional methods, align with the benefits we observed in our work, specifically when measuring turtle carapace dimensions. Additionally, the ability of ImageJ to facilitate detailed analyses of animal structures, including measuring angles between bones, underscores its versatility and applicability across various species and morphological traits and is consistent with our findings regarding turtle carapace measurements.

The broader context of these studies showcases the effectiveness of the ImageJ method in studying animal structures. They consistently demonstrate their capacity to yield accurate and reliable results while enabling in-depth data analyses. Furthermore, the user-friendly and accessible nature of digital images, as highlighted in these studies, reinforces our own observations regarding the advantages of ImageJ in the context of fieldwork and data sharing among researchers. Collectively, these studies emphasize the wide-ranging applications of the ImageJ method in studying the shape and size of animal structures, including sea turtles, fish, and lizards [56–62]. They provide robust evidence supporting the accuracy and reliability of ImageJ in comparison to traditional measurement methods, which is highly relevant to our study’s focus on freshwater turtle morphometric measurements and strengthens the argument for adopting digital methods in animal morphology research. The present study also showcases the decrease in handling time leading to lower stress levels of the animals being measured, besides significantly reducing the effort required to carry out these repetitive but essential measurements for managing distressed animals.

## Conclusion

This study aimed to investigate the reliability and accuracy of two methods, ImageJ and vernier caliper, for measuring the carapace length and width of animals. The findings suggest that both methods are suitable for measuring animal morphology, and the choice of method may depend on various factors, including the complexity of the morphology being measured, the need for high-resolution images, and the availability of equipment. The study demonstrated that ImageJ is a reliable and accurate method for measuring morphology. One of the main benefits of using ImageJ is that it is minimally invasive. ImageJ enables researchers to capture digital images of animal morphology without causing any harm or disturbance to the animal. This is particularly important in fieldwork scenarios where fixed observer and device positioning may be a challenge.

Furthermore, ImageJ offers more flexibility in fieldwork scenarios compared to the vernier caliper method. This is because the digital method allows researchers to capture high-resolution images of the animal morphology, which can be analyzed later in a laboratory setting. In contrast, the vernier caliper method requires the researcher to handle the animal, making it less flexible and more time-consuming. The findings of this study suggest that both ImageJ and vernier caliper methods are accurate and reliable for measuring animal morphology.

In addition, the availability of equipment may also influence the choice of method. The vernier caliper method requires a specialized tool, while ImageJ only requires a digital camera or smartphone. Therefore, if equipment is limited, the ImageJ method may be a more feasible option. In conclusion, this study highlights the importance of selecting appropriate methods for measuring animal morphology. Both methods are suitable for morphometry, and the choice of method may depend on various factors, including the complexity of the morphology being measured, the availability of high-resolution images, and the availability of equipment.

Overall, the methods used in this study are simple, time efficient, low-cost, and suitable for fieldwork, making them a valuable tool for measuring animal morphology. Researchers can use these methods to accurately and reliably measure animal morphology, which can contribute to a better understanding of the ecology, behavior, and evolution of different species. Furthermore, these methods can be easily adapted and applied to a range of different animal species, making them a valuable tool for biologists and ecologists working in different fields.

## References

[1] Gaston KJ, Jackson SF, Cantu-Salazar L, Cruz-Pinon G. The ecological performance of protected areas. Annu Rev Ecology Evolution Syst. 2008;39: 93–113. doi: 10.1146/annurev.ecolsys.39.110707.173529

[2] Hancock PL, Skinner BJ. Desert pavement. The Oxford Companion to the Earth. Oxford University Press; 2000. pp. 1–1.

[3] Dudgeon D, Arthington AH, Gessner MO, Kawabata Z-I, Knowler DJ, Lévêque C, et al. Freshwater biodiversity: importance, threats, status and conservation challenges. Biol Rev. 2006;81: 163–182. doi: 10.1017/S1464793105006950 16336747

[4] Holland RA, Darwall WRT, Smith KG. Conservation priorities for freshwater biodiversity: the key biodiversity area approach refined and tested for continental Africa. Biological Conservation. 2012;148: 167–179. doi: 10.1016/j.biocon.2012.01.016

[5] Abell R, Allan JD, Lehner B. Unlocking the potential of protected areas for freshwaters. Biological Conservation. 2007;134: 48–63. doi: 10.1016/j.biocon.2006.08.017

[6] Thomson SA. Turtles of the world: annotated checklist and atlas of taxonomy, synonymy, distribution, and conservation status. *Phyllomedusa* Journal of Herpetology. 2021;20: 225–228. 10.3854/crm.8.checklist.atlas.v9.2021

[7] Som S, Cottet M. Rescue and relocation programme of turtles and tortoises and elongated tortoise monitoring programme in the Nam Theun 2 Reservoir (Laos). *Hydroécologie Appliquée*. 2016;19: 383–406. doi: 10.1051/hydro/2015007

[8] Badola S, Choudhary AN, Chhabra DB. Tortoises and Freshwater Turtles in illegal trade in India. TRAFFIC India New Delhi. 2019.

[9] Vasudevan K. Freshwater Turtles and Tortoises of India. ENVIS Bull Wildlifeand Protected Areas. 2009;12.

[10] Chandrakar AK, Yadav KK, Kumar V, Gupta N. Scenario of biodiversity conservation in India: An overview. 2016.

[11] Mast RB, Mittermeier CG, Mittermeier RA, Rodríguez-Mahecha JV, Hemphill AH. Megadiversity: Earth‘s Biologically Wealthiest Nations. Mittermeier RA Robles Gil P Mittermeier CG Eds. 1997; 314–324.

[12] Verma A, Trivedi R. Fresh Water Biodiversity-Challenges from Indian Perspective and Conservational Approaches Chapter 8. 2016. pp. 139–153.

[13] Stanford CB, Iverson JB, Rhodin AG, van Dijk PP, Mittermeier RA, Kuchling G, et al. Turtles and tortoises are in trouble. Curr Biol. 2020;30: R721–R735. doi: 10.1016/j.cub.2020.04.088 32574638

[14] Fund TC. A global action plan for conservation of tortoises and freshwater turtles. Strategy Funding Prospectus. 2002;2007: 30.

[15] Cope HR, McArthur C, Dickman CR, Newsome TM, Gray R, Herbert CA. A systematic review of factors affecting wildlife survival during rehabilitation and release. PLoS One. 2022;17: e0265514. doi: 10.1371/journal.pone.0265514 35298527 PMC8929655

[16] Talukdar A, Panda A, Srivastav A, Hussain SA, Malik PK, Nigam P. Growth patterns of critically endangered, head-started three-striped roofed turtle, *Batagur dhongoka* (Gray, 1834). Biologia (Bratisl). 2021;76: 3705–3710. doi: 10.1007/s11756-021-00858-y

[17] Terentjev PV, Chernov SA. Guide to reptiles and amphibians. Sov Prees Mosc Leningr. 1949.

[18] Pritchard P, Bacon PR, Berry FH, Carr AF, Fletemeyer J, Gallagher RM, et al. Protecting nesting beaches. Man Sea Turtle Resarch and Conservtion Techniques. 1983; 85–88.

[19] Perälä J. A new species of Testudo (Testudines: Testudinidae) from the Middle East, with implications for conservation. J Herpetol. 2001; 567–582. doi: 10.2307/1565894

[20] Conn BPB, Alisauskas RT. Simultaneous Modelling of Movement, Measurement Error, and Observer Dependence in Double-Observer Distance Sampling Surveys. bioRxiv. 2017; 126821. doi: 10.1101/126821

[21] Duijns S, Knot IE, Piersma T, van Gils JA. Field measurements give biased estimates of functional response parameters, but help explain foraging distributions. J Anim Ecol. 2015;84: 565–575. doi: 10.1111/1365-2656.12309 25327649

[22] Gertsbakh I, Gertsbakh I. Introduction: Measurand and Measurement Errors. Meas Theory Eng. 2003; 1–8.

[23] Plyusnin I, Evans AR, Karme A, Gionis A, Jernvall J. Automated 3D phenotype analysis using data mining. PLoS One. 2008;3: e1742. doi: 10.1371/journal.pone.0001742 18320060 PMC2254194

[24] Castleman K.R., 1996. Digital image processing. Prentice Hall Press.

[25] Meijering E, Cappellen GV. Quantitative Biological Image Analysis. In: Shorte SL, Frischknecht F, editors. Imaging Cellular and Molecular Biological Functions. Berlin, Heidelberg: Springer Berlin Heidelberg; 2007. pp. 45–70. doi: 10.1007/978-3-540-71331-9_2

[26] Pham DL, Xu C, Prince JL. Current Methods in Medical Image Segmentation. Annu Rev Biomed Eng. 2000;2: 315–337. doi: 10.1146/annurev.bioeng.2.1.315 11701515

[27] Sluder G, Wolf DE, editors (2007) Digital Microscopy, Volume 81: Methods in Cell Biology San Diego, CA: Academic Press. 343–347.

[28] Schindelin J, Arganda-Carreras I, Frise E, Kaynig V, Longair M, Pietzsch T, et al. Fiji: an open-source platform for biological-image analysis. Nat Methods. 2012;9: 676–682. doi: 10.1038/nmeth.2019 22743772 PMC3855844

[29] Claude J, Paradis E, Tong H, Auffray J-C. A geometric morphometric assessment of the effects of environment and cladogenesis on the evolution of the turtle shell. Biol J Linn Soc. 2003;79: 485–501. doi: 10.1046/j.1095-8312.2003.00198.x

[30] Roth VL, Mercer JM. Morphometrics in development and evolution. Am Zool. 2000;40: 801–810. doi: 10.1093/icb/40.5.801

[31] Talukdar A, Sengupta D, Mallapur G, Hussain SA, Malik PK, Nigam P. Amelioration of the Freshwater Turtle Breeding and Rehabilitation Station in Varanasi, India. Reptil Amphib. 2019;26: 170–173.

[32] Eckert KL, Bjorndal KA, Abreu-Grobois FA, Donnelly M. Techniques for measuring sea turtles. Measurements. 1986;1988.

[33] Gerosa G, Aureggi M. Sea turtle handling guidebook for fishermen–teaching book. UNEP/MAP RAC/SPA, Tunis, Tunisia. 2001.

[34] Schneider CA, Rasband WS, Eliceiri KW. NIH Image to ImageJ: 25 years of image analysis. Nat Methods. 2012;9: 671–675. doi: 10.1038/nmeth.2089 22930834 PMC5554542

[35] Cagle FR. A system of marking turtles for future identification. Copeia. 1939;1939: 170–173. doi: 10.2307/1436818

[36] Moore DS. Introduction to the Practice of Statistics. WH Freeman and company; 2009.

[37] Chai T, Draxler RR. Root mean square error (RMSE) or mean absolute error (MAE). Geosci Model Dev Discuss. 2014;7: 1525–1534. 10.5194/gmdd-7-1525-2014

[38] Gahm NA, Rueden CT, Evans EL III, Selzer G, Hiner MC, Chacko JV, et al. New extensibility and scripting Tools in the ImageJ ecosystem. Curr Protoc. 2021;1: e204. doi: 10.1002/cpz1.204 34370407 PMC8363112

[39] Song Y, Qin Z, Rong X. Approach to detect image edge and prospect by imagej. Mech Manag Dev. 2008;23: 180–181.

[40] Chiari Y, Wang B, Rushmeier H, Caccone A. Using digital images to reconstruct three-dimensional biological forms: a new tool for morphological studies. Biol J Linn Soc. 2008;95: 425–436. doi: 10.1111/j.1095-8312.2008.01055.x

[41] Coorens NA, Daemen JH, Slump CH, Loonen TG, Vissers YL, Hulsewé KW, et al. The automatic quantification of morphological features of pectus excavatum based on three-dimensional images. Seminars in Thoracic and Cardiovascular Surgery. Elsevier; 2022. pp. 772–781. 10.1053/j.semtcvs.2021.05.018 34102293

[42] Mahendiran M, Parthiban M, Azeez PA, Nagarajan R. In situ measurements of animal morphological features: A non-invasive method. Methods Ecol Evol. 2018;9: 613–623. doi: 10.1111/2041-210X.12898

[43] Jensen EC. Quantitative analysis of histological staining and fluorescence using ImageJ. Anat Rec. 2013;296: 378–381. doi: 10.1002/ar.22641 23382140

[44] Rueden CT, Schindelin J, Hiner MC, DeZonia BE, Walter AE, Arena ET, et al. ImageJ2: ImageJ for the next generation of scientific image data. BMC Bioinformatics. 2017;18: 1–26. doi: 10.1186/s12859-017-1934-z 29187165 PMC5708080

[45] Bılıcı S, Kaya A, Cıcek T, Dörtbudak MY. Investigation of size and shape differences depend to sex, age and season on scales of smallmouth lotak (*Cyprinion kais*). Surv Fish Sci. 2016;3: 37–45.

[46] Hua R, Lia F, Yua H, Yanga J. Application of ImageJ in the rock thin section image analysis: the separation and quantitative calculation of crystal-glass two phases. https://DOI.org/10.25177/JESES.4.3.RA.502

[47] Trabuco JR, Martins SAM, Prazeres DMF. Use of ImageJ to Recover Information from Individual Cells in a G Protein-Coupled Receptor Assay. G Protein-Coupled Recept Screen Assays Methods Protoc. 2015; 143–172. doi: 10.1007/978-1-4939-2336-6_11 25563183

[48] Haq RU. Environmental Studies Of Different Effects Of Lead On Some Physiological And Morphological Features Of Diptera Flies. PhD Thesis, Federal urdu university of arts, science and technology, gulshan-e-iqbal, 2012.

[49] Onder H, Arı A, Ocak S, Eker S, Tufekci H. Use of image analysis in animal science. J Inf Techol Agric. 2011;1: 1–4.

[50] Dalton HA, Wood BJ, Widowski TM, Guerin MT, Torrey S. An analysis of beak shape variation in two ages of domestic turkeys (Meleagris gallopavo) using landmark-based geometric morphometrics. PLoS One. 2017;12: e0185159. doi: 10.1371/journal.pone.0185159 28934330 PMC5608350

[51] Taravati S, Darvish J, Mirshamsi O. Geometric morphometric study of two species of the psammophilous genus Erodiontes (Coleoptera: Tenebrionidae) from the Lute desert, Central Iran. Iran J Anim Biosyst. 2009;5. Available: https://ijab.um.ac.ir/article/view/article_25222.html doi: 10.22067/IJAB.V5I2.3341

[52] Moneva CSO, Demayo CG, Torres MAJ. Applications of geometric morphometric analysis in describing sexual dimorphism in shell shapes in Vivipara angularis Muller (Family Viviparidae). Anim Biol Anim Husb. 2012;4: 14–19.

[53] Klingenberg CP. Morpho J: an integrated software package for geometric morphometrics. Mol Ecol Resour. 2011;11: 353–357. doi: 10.1111/j.1755-0998.2010.02924.x 21429143

[54] Gordillo S, Márquez F, Cárdenas J, Zubimendi MÁ. Shell variability in Tawera gayi (Veneridae) from southern South America: a morphometric approach based on contour analysis. J Mar Biol Assoc U K. 2011;91: 815–822. doi: 10.1017/S0025315410000391

[55] Moreddu E, Puymerail L, Michel J, Achache M, Dessi P, Adalian P. Morphometric measurements and sexual dimorphism of the piriform aperture in adults. Surg Radiol Anat. 2013;35: 917–924. doi: 10.1007/s00276-013-1116-2 23625070

[56] Maulida FF, Imron MA, Reischig T. Geometry morphometry and health status of hawksbill turtle (*Eretmochelys imbricata* Linnaeus, 1766) in Maratua Island, East Kalimantan-Indonesia. KnE Life Sci. 2017; 100–110. doi: 10.18502/kls.v3i4.693

[57] Zhang Y, Miao G, Fazhan H, Waiho K, Zheng H, Li S, et al. Transcriptome-seq provides insights into sex-preference pattern of gene expression between testis and ovary of the crucifix crab (*Charybdis feriatus*). Physiol Genomics. 2018;50: 393–405. doi: 10.1152/physiolgenomics.00016.2018 29570432

[58] Mojekwu TO, Anumudu CI. Advanced techniques for morphometric analysis in fish. J Aquac Res Dev. 2015;6: 1–6.

[59] Samara B, Deliorman M, Sukumar P, Qasaimeh MA. Cryopreservable arrays of paper-based 3D tumor models for high throughput drug screening. Lab Chip. 2021;21: 844–854. doi: 10.1039/d0lc01300e 33615319

[60] Stayton CT. Performance in three shell functions predicts the phenotypic distribution of hard-shelled turtles. Evolution. 2019;73: 720–734. doi: 10.1111/evo.13709 30820948

[61] Schmidt BV, Schaefer J. Ecological and landscape effects on genetic distance in an assemblage of headwater fishes. Ecol Freshw Fish. 2018;27: 617–631. doi: 10.1111/eff.12375 10.1111/eff.12375

[62] Llewelyn J, Macdonald S, Hatcher A, Moritz C, Phillips BL. Thermoregulatory behaviour explains counter gradient variation in the upper thermal limit of a rainforest skink. Oikos. 2017;126: 748–757. doi: 10.1111/oik.03933

